# Our Evolving Understanding of the Mechanism of Quinolones

**DOI:** 10.3390/antibiotics7020032

**Published:** 2018-04-08

**Authors:** Arnaud Gutierrez, Jonathan M. Stokes, Ivan Matic

**Affiliations:** 1 Inserm U1001, Université Paris Descartes, Sorbonne Paris Cité, Faculté de Médecine Paris Descartes, 75014 Paris, France; gutierrez.arnaud@gmail.com; 2 Institute for Medical Engineering & Science, Department of Biological Engineering, and Synthetic Biology Center, Massachusetts Institute of Technology, Cambridge, MA 02139, USA; stokesjm@mit.edu; 3 Infectious Disease and Microbiome Program, Broad Institute of MIT and Harvard, Cambridge, MA 02142, USA; 4 Department of Life Sciences, Centre National de la Recherche Scientifique, 75016 Paris, France

**Keywords:** antibiotics, quinolones, topoisomerases, DNA replication, DNA supercoiling

## Abstract

The maintenance of DNA supercoiling is essential for the proper regulation of a plethora of biological processes. As a consequence of this mode of regulation, ahead of the replication fork, DNA replication machinery is prone to introducing supercoiled regions into the DNA double helix. Resolution of DNA supercoiling is essential to maintain DNA replication rates that are amenable to life. This resolution is handled by evolutionarily conserved enzymes known as topoisomerases. The activity of topoisomerases is essential, and therefore constitutes a prime candidate for targeting by antibiotics. In this review, we present hallmark investigations describing the mode of action of quinolones, one of the antibacterial classes targeting the function of topoisomerases in bacteria. By chronologically analyzing data gathered on the mode of action of this imperative antibiotic class, we highlight the necessity to look beyond primary drug-target interactions towards thoroughly understanding the mechanism of quinolones at the level of the cell.

## 1. Introduction

Nine decades after the discovery of penicillin, antibiotics remain our primary tool to combat bacterial infections. However, after a period of highly productive drug discovery spanning from the 1940s to the 1960s, substantially fewer molecules are now reaching the clinic, referred to as the so-called “discovery void”, which has persisted since the 1990s. The constant evolution and dissemination of antibiotic resistance determinants is becoming a serious threat to our ability to effectively treat bacterial infections [1]. In order to maximize the efficacy of our current antibiotic arsenal, it is imperative to develop a thorough understanding of the mode of action of these molecules in the context of the cell, as well as the extracellular factors that may influence their efficacy.

Examination of antibiotic target diversity in the context of our current clinical antibiotics reveals that the number of macromolecular processes targeted is limited to cell wall synthesis, translation, transcription, and DNA replication. Among drugs affecting DNA replication, the antibiotic classes that target DNA topology, such as the quinolones, are an essential part of our clinical antibiotic panel. However, as with all antibiotics, quinolone efficacy is threatened by the consistent rise of resistant strains isolated from clinical and agricultural settings [2,3]. Remarkably, despite our reasonable understanding of the quinolone targets and the mechanisms of resistance, there is a conspicuous lack of understanding of how precisely this family of antibiotics results in bacterial cell killing.

In this review, we examine some of the early literature that probed the mode of action of quinolones and interpret these data in the context of modern investigations. Specifically, we highlight the fact that long before the targets of quinolones were discovered, studies on the mechanism of these antibiotics revealed the critical role of bacterial cell metabolism in their ability to promote cell death. From this perspective, we posit that an appreciation of the biological processes distal to primary target corruption can significantly help to optimize our antibiotic applications in the clinic.

## 2. The Molecular Target of Quinolones: Type II Topoisomerases

The structure and topology of the genome is constrained by the nature of the double-stranded DNA helix [4]. Thus, any processes that require opening of the double helix can be facilitated by the introduction of negative supercoiling, while the continual unwinding of the DNA helix during replication will simultaneously promote positive supercoiling [5,6]. Modification of DNA supercoiling is a critical event during cell cycle progression, as it is essential for the accurate DNA binding activity of transcription regulators, and is similarly well suited to modulate essential processes such as DNA replication and transcription [7,8]. The resolution of DNA topological constrains requires (i) breaking of the sugar-phosphate backbone, and (ii) the migration of DNA strands toward the break. To resolve these topological conflicts, cells use conserved enzymes called topoisomerases [9], which are classified into two groups: type I, which promote single strand nicking of DNA; and type II, which produce double-strand breaks. Bacterial DNA topoisomerase II, which includes the structurally related DNA gyrase and topoisomerase (topo) IV, are essential and conserved enzymes that differ sufficiently from their eukaryotic homologs to be a prime candidate for antibiotic targeting [10]. Of note, type II topoisomerases can be further divided into the IIA and IIB subclasses based on biochemical and structural divergence. Type IIB enzymes are found exclusively in archaea and plants, and therefore all further discussions will pertain specifically to type IIA topoisomerases. Most bacteria possess two type II topoisomerases, namely gyrase and topo IV, with a few exceptions, such as Mycobacteria that possess only gyrase. Both enzymes process DNA by promoting the formation of a transient protein-DNA crosslink, the cleavage complex, wherein the 5′ end of cleaved DNA becomes bound to a conserved tyrosine residue on the protein. As such, an ATP dependent process, termed a two-gate mechanism, promotes the topological change [11,12,13], followed by a rapid re-ligation step (structural and biochemical mechanisms are reviewed in [9]). While numerous classes of molecules, such as aminocoumarins and the peptide CcdB, can interact with and affect topoisomerase function, we will primarily focus on the clinically imperative quinolone class of antibiotics in forthcoming discussions.

Quinolones specifically corrupt type IIA DNA topoisomerases by preventing the re-ligation step of topology correction, thereby trapping the cleavage complex on DNA and ultimately promoting cell death [14]. The discovery of quinolones began with the isolation of nalidixic acid in the early 1960s [10,15]. Molecules from the first quinolone generation were, at this time, primarily used for the treatment of urinary tract infections. Starting in the early 1980s, medicinal chemistry efforts to modify the quinolone backbone led to the synthesis of the 2nd, 3rd and 4th generations of compounds, namely fluoroquinolones [16]. Compared to 1st generation compounds, fluoroquinolones possessed improved pharmacological properties, with a lower minimum inhibitory concentration (MIC), greater tissue penetration, and a broader spectrum of susceptible pathogens [14,16]. Notably, fluoroquinolones are currently one of the primary antibiotic families prescribed [17].

From their initial discovery, significant efforts have been made to understand the mechanism of action of quinolones [18]. Interestingly, quinolone targets, namely DNA gyrase and topo IV, were discovered 16 and 27 years after the first introduction of nalidixic acid, respectively. Remarkably, however, during the “pre-target” era, much of the pertinent information related to quinolones mechanism of action had been elucidated [19,20,21,22,23,24]. We present these findings below and discuss them in the context of more modern observations pertaining to the details of quinolone bioactivity.

## 3. Quinolone Mechanism Prior to Target Identification

Many sophisticated concepts related to the mode of action of quinolones were described prior to the discovery of their specific intracellular binding partners. One of the first striking properties of the 1st generation quinolones was that their antibacterial activity was only effective on proliferating cells, as their bactericidal effect was inhibited under suboptimal growth conditions [23]. Indeed, growth-arrested bacterial populations with low temperatures or nitrogen starvation were not susceptible to killing by nalidixic acid. Examination of exponentially growing bacterial cultures revealed that nalidixic acid promoted a reduction of viability without a corresponding loss of culture turbidity. Rather, using microscopy, it was observed that nalidixic acid-treated cultures displayed dose-dependent cellular filamentation [23]. Importantly, this treatment-induced filamentation did not increase osmotic sensitivity, nor promote membrane damage. These observations differentiated quinolones mechanism from that of the penicillins, which were also known to cause filamentation.

Based on these observations, it was evident that the metabolic state of the cell was a significant determinant for the bactericidal activity of quinolones [23]. Because cell growth and active protein synthesis were found to be required for nalidixic acid bactericidal activity, DNA replication was proposed to be targeted by quinolones. This was subsequently confirmed by analyzing the rates of accumulation of radiolabeled nucleotides in nalidixic acid-treated cells [24]. Through these approaches, it was observed that nalidixic acid treatment reversibly inhibited DNA synthesis in a dose dependent manner, within 10 min (about 0.5 doublings) of treatment, while protein and RNA synthesis were minimally affected. Furthermore, as early as 1965, using the multi-auxotrophic *Escherichia coli* TAU (Thymine, Arginine, Uracil strain [25], it was shown that nalidixic acid-induced lethality was dependent on RNA and protein synthesis [20,22,24]. Indeed, nalidixic acid treatment can halt DNA replication in amino acid or uracil-starved cells without promoting cell death [24]. Furthermore, nalidixic acid treatment can promote DNA synthesis arrest in chloramphenicol-pretreated cells without causing death [22], showing that DNA synthesis inhibition is likely caused by a direct interaction between the quinolone and DNA, or the quinolone and enzymes involved in the DNA replication processes. Because DNA replication arrest is reversible, and because bacterial cultures can be rescued by washing [22,24], it was proposed that the interaction was either weak or reversible. As a consequence of the reversible nature of nalidixic acid activity, and since its bactericidal action required RNA or protein synthesis, it was hypothesized that the bactericidal activity of nalidixic acid was dependent on the metabolic activity of the cells [23,24]. This was tested by the treatment of bacterial cultures with dinitrophenol [22], which is an electron transport chain uncoupler that impairs cellular respiration. Treatment with dinitrophenol protected cell populations treated with bactericidal concentrations of nalidixic acid more efficiently than did chloramphenicol. This provided further evidence that the bactericidal action of nalidixic acid was directly related to the metabolic state of the treated cells [22].

The first suggestions of the critical events triggering cell death by nalidixic acid came after studying the stability of cellular components, namely DNA, RNA and proteins [20]. The bactericidal activity of nalidixic acid was proposed to coincide with DNA degradation, as this was strongly correlated with the percentage of viable cells. As a result, the mode of action of quinolones was described as follows: “Nalidixic acid rapidly inhibits DNA synthesis in growing cells of *E. coli*. This inhibition renders the DNA of such cells vulnerable to attack by endogenous nucleases, and the genetic material is ultimately destroyed” [20]. We would like to emphasize that here, the observed DNA degradation was dependent on active metabolism, RNA transcription, and protein synthesis, and therefore could result from metabolism-dependent DNA damage and/or DNA digestion by intracellular nucleases. Because nalidixic acid did not directly damage DNA or impair DNA polymerase [19], the primary question was then to elucidate the processes through which quinolones were able to inhibit DNA replication and promote subsequent DNA degradation.

Importantly, while quinolones such as nalidixic acid were initially shown to have no activity against protein or RNA synthesis, re-examination at higher drug concentrations showed the opposite phenotype [20,26]. Indeed, concentrations of nalidixic acid greater than 10-fold MIC were observed to inhibit protein translation, and concentrations greater than 50-fold MIC could inhibit RNA synthesis [27]. Notably, concentrations that inhibited protein synthesis correlated with a reduction in the killing efficiency of the drug [26].

## 4. The Discovery of DNA Gyrase and Topo IV as the Targets of Quinolones

DNA gyrase, the primary target of quinolones in Gram-negative bacteria, was first reported in 1976 [28]. Gyrase activity was shown to catalyze the ATP-dependent formation of thermodynamically unfavorable negative supercoiled DNA, a function that appeared to be essential for plasmid replication and regulation of phage recombination. Soon after the elucidation of gyrase activity, mutant resistant to quinolones were associated with its topoisomerase function [29,30]. Therefore, since the DNA supercoiling activity of purified gyrase from nalidixic acid-resistant mutants showed cross-resistance to the action of oxolinic acid, it was concluded that quinolones were able to inhibit the nicking–resealing activity of DNA gyrase. A second type IIA topoisomerase targeted by quinolones, DNA topo IV, was reported in *E. coli* in 1990 [31]. Both enzymatic complexes shared the same mode of action, but possessed unique functions. In Gram-negative bacteria, gyrase is primarily involved in the resolution of the topological constraint produced by DNA replication, while topo IV is responsible for the resolution of catenated DNA [9].

The discovery of the intracellular binding partner of quinolones brought new tools for the understanding of the detailed mode of their bactericidal action in the context of the cell. Indeed, examination of chromosome sedimentation profiles showed that inhibition of DNA gyrase by oxolinic acid did not directly promote the formation of free DNA ends. Rather, DNA breaks could be released upon the addition of detergent [32]. This observation, together with the observations that (a) the nalidixic acid resistant allele Nal^R^ was recessive over Nal^S^ [33]; (b) that DNA replication of a strain bearing a thermosensitive *gyrA* allele (nalA45^ts^) was not impaired by nalidixic acid [34]; and (c) that T7 phage replication, which does not strictly required gyrase, could be arrested in a GyrA-dependent manner by nalidixic acid treatment [34], together led to the hypothesis that quinolones corrupted the DNA gyrase complex, rather than simply prevented its function.

The purification of DNA gyrase similarly allowed for a significantly more thorough biochemical understanding of topoisomerase/quinolone activity. In vitro biochemical assays provided strong evidence that the topoisomerase activity was an energetic process requiring ATP consumption, and suggested that the energetic status of cells represented by the ratio between ATP and ADP (adenylate energy charge) could greatly influence DNA supercoiling [35]. These data additionally predicted that rapid transition in growth conditions, such as carbon diauxy, aero-anaerobic transition, or osmotic shock, all of which affect ATP/ADP ratio, could modulate gyrase activity and DNA supercoiling [8,36,37,38,39]. Through investigations into how the energy status of the cell could affect gyrase activity, it was reported that DNA supercoiling could modulate gene expression [40,41], and that the degree of negative supercoiling could impact protein abundance either positively or negatively [42]. Remarkably, most of the genes that were modulated by DNA supercoiling were related to metabolic processes and were involved in virulence [43]. We believe that this observation is currently overlooked, and could be crucial towards understanding the environmental conditions that modulate quinolone activity, and how different concentrations of quinolones can lead to discrete cell states that are more or less susceptible to subsequent antibiotic-dependent eradication [44,45].

## 5. Quinolone-Induced DNA Breaks

In 1966, it was demonstrated that nalidixic acid activity ultimately triggered the degradation of DNA [20], and that recombination-deficient mutants were hypersensitive to quinolones [46], thereby indicating the formation of double-stranded breaks in vivo. However, the specific sequence of molecular events leading to double-stranded break formation and degradation of DNA is still far from clear. First, investigators found evidence of the generation of double-stranded DNA breaks induced by quinolone treatment [7]. For some time, the conceptual challenge was to link the formation of a covalently trapped cleavage complex to double-stranded DNA breaks. One of the earliest models proposed was that the trapped complex could result in double-stranded DNA breaks through collision with the DNA replication complex (reviewed in [14,18]). This was largely ruled out using in vitro assays [47] and by the use of a temperature-sensitive *dnaB*^ts^ mutant, which impairs DNA replication at non-permissive temperatures [48]. This model was refined to encompass chromosome fragmentation induced by destabilization of the cleavage complex, occurring mainly during DNA gyrase interaction with the later generation quinolones, such as gatifloxacin [49]. An alternative, but not mutually exclusive, mechanism proposed the formation of double-stranded DNA breaks to be a result of collateral damage [50]. While the trapped cleavage complex halts DNA replication fork progression, here it is proposed that the processing of blocked and regressed DNA replication forks by endonucleases RuvC or SbcCD will result in breaking DNA [51]. While the exact sequence of events can still be significantly refined, it is evident that quinolones promote the formation of DNA breaks, as it has been visualized in vivo [52]. An interesting question to us is not where the DNA break is appearing, whether at the cleavage complex site or at an alternative position, but why repair proficient cells are not able to process and repair such DNA breaks. The obvious solution is that the number of DNA breaks may simply be too large to be repaired, but it could also be that the breaks might be formed in a manner rendering them less amenable for repair. For example, if formed at the cleavage complex, cross-linked topoisomerase subunits could sterically hinder access by the nucleases required to process DNA for double-stranded break repair via homologous recombination.

## 6. Beyond Target Inhibition: Metabolism, Respiration and Oxidative Stress

Relatively recent access to large-scale quantitative measurements such as gene microarrays and RNA sequencing has helped to popularize the notion that cellular death promoted by antibiotics, including quinolones, is rooted in their ability to promote metabolic perturbation downstream of the corruption of the primary target [53,54,55]. Gene expression analyses indicate that quinolone treatment induces a change in the transcription of genes coding for central carbon metabolism and oxidative respiration enzymes [56]. Quinolone-induced changes in metabolic homeostasis were subsequently proposed to lead to the formation of promiscuously reactive molecules, such as reactive oxygen species (ROS) and reactive nitrogen species. This notion was later confirmed by genetic evidence, such as the hypersensitivity of different oxidative repair deficient mutants to quinolones [57,58]. The direct implication of ROS in quinolone lethality was challenged by two publications ([59,60]; see also two recent reviews [55,61]). However, we would like to underline that since the early studies on the mode of action of quinolones, it has been clear that cellular respiration and metabolism are key factors influencing the fate of bacteria challenged by these molecules [23,24]. Indeed, protection of the bactericidal action of quinolones induced by transcription and translation inhibitors was recently explained by their direct effect on cellular respiration [62]. The metabolic state of cells challenged by quinolones seems, therefore, to be essential for determining the bioactivity of the drug. For example, the cellular metabolic state alters topoisomerase activity by tuning the adenylate energy charge ratio. A more thorough understanding of how environmental conditions modulate cellular metabolic state, and how these, in turn, modulate quinolone activity, may help identify mechanisms to sensitize bacteria to the action of quinolones. Indeed, it is possible to sensitize high cell density cultures to quinolones by promoting respiration, and through supplementation of a suitable electron acceptor and carbon sources [63].

## 7. Conclusions

Beyond providing a brief historical overview of the mechanism of quinolones, we highlight that the identification of the intracellular binding partner of antibiotics may not be sufficient to explain all antibiotic-induced biological effects. The example of quinolones underlines the critical role of bacterial cell metabolic processes in the ability of these molecules to promote cell death. Indeed, this was acknowledged long before gyrase and topo IV had been discovered and shown to bind quinolones. Unfortunately, upon elucidation of the intermolecular interaction between quinolones and their targets, this knowledge of the importance of metabolic state was ignored—it was considered largely irrelevant in describing the lethality of quinolones. However, modern systems-level approaches are highlighting the importance of understanding antibiotic activity in the greater context of the cell as a whole, working towards the development of therapeutic strategies that maximize the efficacy of our antibiotic arsenal in the face of overwhelming resistance.
